# Supplementary material for: Contrasting genes conferring short- and long-term biofilm adaptation in Listeria
Source: Microb Genom. 2023 Oct 18;9(10):001114. doi: 10.1099/mgen.0.001114 (PMC10634452; doi:10.1099/mgen.0.001114)
Supplement: Supplementary material 1 [file mgen-9-1114-s001.pdf]

Supplementary methods

CheckM (version 1.0.18) (Parks *et al.*, 2015) was used to provide estimates of genome assembly completeness and contamination. **Table S7** shows the completeness and contamination metrics for 27 *L. monocytogenes* genome assemblies that contain more than 1000 contigs. Assemblies with <90% completeness or >5% contamination were removed (*n*=12) from the analysis and downstream analyses were performed on this reduced dataset.

Table S7.

id	completeness	contamination
**26981**	95.29	3.15
**26951**	96.82	4.83
**26906**	97.22	0.65
**26916**	95.9	4.35
**26979**	95.81	4.16
**26931**	94.11	4.92
**26943**	90.16	3.24
**26900**	95.84	2.6
**26980**	97.15	3.35
**26936**	92.86	4.82
**26901**	86.27	4.89
**26902**	93.55	3.03
**26945**	87.27	4.26
**26913**	91.67	4.78
**26971**	93.87	4.29
**26982**	98.28	4.57
**26947**	99.45	4.62
**26958**	82.97	0.62
**26890**	77.09	1.45
**26926**	84.57	2.84
**26969**	87.56	4.33
**26928**	84.39	8.29
**26964**	88.54	6.28
**26944**	70.71	3.06
**26894**	73.94	3.31
**26929**	65.48	1.84
**26934**	failed	failed

Concatenated core genome alignments were produced gene-by-gene using MAFFT (*n*=96, 1642 genes, 1607055 bp) and a maximum likelihood phylogeny was inferred from the

alignment using RAxML (version 8.2.4) (Stamatakis, 2014) with the generalized time-reversible (GTRGAMMA) substitution model. The resulting phylogeny and core-genome alignment was used as input to ClonalFrameML (version 1.12) (Didelot and Wilson, 2015). GWAS was performed using Pyseer (version 1.3.6) (Lees *et al.*, 2018), as previously described, to test genetic variation for its association with biofilm formation at 30°C and 37°C. A genetic relatedness matrix was calculated from the inferred population phylogeny of the reduced dataset and this was used to apply a phylogenetic correction to adjust the *p*-value of association variants. Gene function was inferred by mapping variants to the *L. monocytogenes* F2365 complete reference genome (assembly accession: NC_002973.6) using BWA-MEM (version 0.7.17) (Li, 2013).

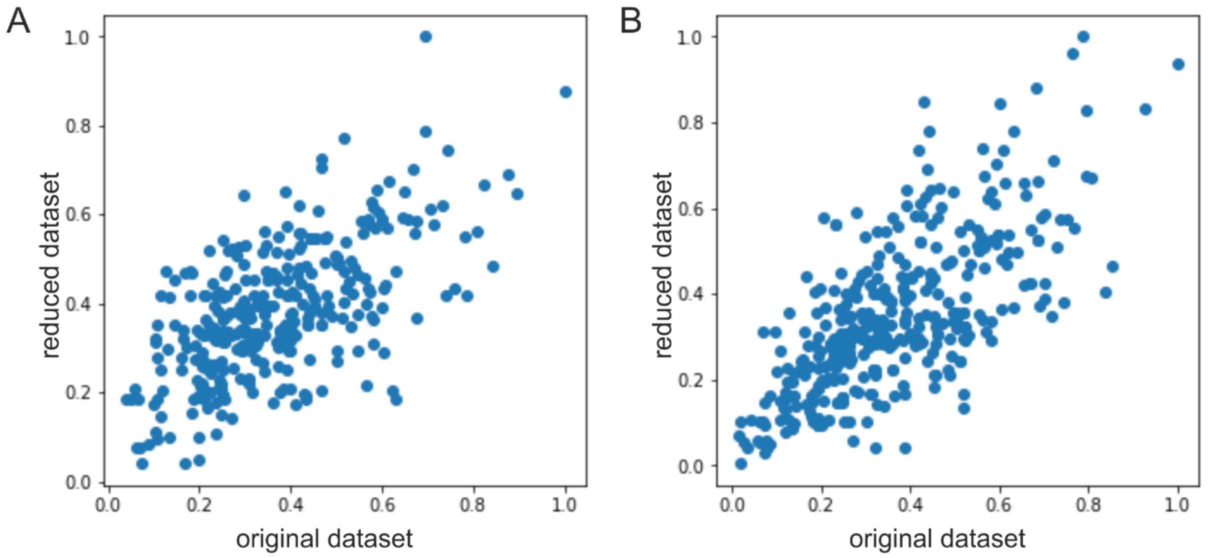

Supplementary figure 1: GWAS results using a reduced dataset correlate with original GWAS results. (A) *p*-values of 460 genes associated with biofilm formation at 30°C. (B) *p*-values of 403 genes associated with biofilm formation at 37°C. *p*-values are shown as a proportion of the maximal *p*-value per experiment. Only genes associated with biofilm formation in both the original GWAS and reduced GWAS analyses are included.
